# Longitudinal prediction of primary school children’s COVID-related future anxiety in the second year of the pandemic in Germany

**DOI:** 10.1371/journal.pone.0302065

**Published:** 2024-05-08

**Authors:** Katharina Voltmer, Maria von Salisch

**Affiliations:** Institute for Sustainability Psychology, Leuphana University Lueneburg, Lueneburg, Lower Saxony, Germany; University of Bristol, UNITED KINGDOM

## Abstract

Although research has confirmed that the first COVID-19-related lockdown has increased stress and mental health problems in children, less is known about the longer-term effects of the pandemic on children’s COVID-related future anxiety (CRFA). Because of CRFA’s potentially debilitating effects, risk and resilience factors against this anxiety were investigated. To this end, *n* = 140 children (49% female) in 3^rd^ and 4^th^ grade classrooms in Germany were asked to perform a working memory task and to self-report about their CRFA and emotion regulation in December 2020 and in May 2021. More maladaptive emotion regulation in December 2020 contributed to the explanation of a high CRFA score in May 2021, whereas a better performance on working memory updating contributed a lower CRFA score later when controls were in place. These results were confirmed when children’s CRFA in December 2020 was included in the prediction of their later CRFA. They suggest that maladaptive strategies of emotion regulation, such as rumination, may explain higher or increasing levels of CRFA, whereas efficient working memory updating may be an indicator of processing information in a way which shields children from CRFA-related thoughts. The concepts underlying these variables should be included in prevention and intervention efforts.

## Introduction

The COVID-19 pandemic and the ensuing lockdown in March to June 2020 have adversely affected children’s mental health world-wide [1, 2]. According to a large online study, 59% of the primary school children in Germany felt “rather” or “clearly” stressed, irritated, or lonely, mostly because they could not go out to play or to meet their friends [3].Other online studies have noted an increase in negative emotions [4] and a decrease in adaptive forms of emotion regulation in children [5] over the course of the first lockdown in Europe. In fact, children’s and adolescents’ mental health deteriorated in many countries when compared to pre-pandemic levels [6–8] (e.g., Bhogal et al., 2021; Hussong et al., 2021; Madigan et al. 2023). In the representative sample of the German Corona and Psyche (COPSY) study, for example, significantly more 7- to 17-year-olds experienced borderline or abnormal mental health problems than an equally representative sample before the pandemic. Anxiety symptoms in the clinical range likewise increased when compared to before the pandemic [9].

Whereas increasing levels of anxiety were to be expected during the uncertainty and the social isolation of the first lockdown, less is known about the longer-term effects of the pandemic on children’s mental health. One of the exceptions is the German COPSY study which followed a representative sample of 1586 families with children from the first lockdown in April 2020 to a second data collection during the second lockdown between December 2020 and January 2021 and a third during regular school attendance in the fall of 2021. Symptoms of generalized anxiety captured by the Screen for Child Anxiety Related Emotional Disorders [10] increased from 24% during the first lockdown to 30% during the second lockdown and decreased slightly to 27% in the fall of 2021 [11]. The high level of anxiety during the first lockdown and the increase towards the second lockdown were corroborated by the systematic review of longitudinal studies by Miao et al. [12]. Because children in Germany stayed at home during the first and the second lockdown, effects of social isolation due to school closures may have increased their anxiety [13]. Nevertheless, their anxiety level has not returned to pre-pandemic levels even after 18 months of the pandemic [11]. The timing of the measurement in relation to the phases of the pandemic therefore seems to be of paramount importance.

### COVID-related future anxiety

The future can be seen as a space full of opportunities, as a space of uncertainty or as something in between. Zaleski [14] defined future anxiety as a "state of apprehension, uncertainty, fear, worry, and anxiety about unfavorable changes in a more distant personal future" [14]. According to Zaleski [14], future anxiety is caused by cognitive representations of individuals’ more distant future, in which they are aware of future events that they will have to deal with and whose consequences they will have to endure. The mental engagement with the distant future distinguishes future anxiety from other anxieties in which, for example, direct physical confrontation with a (very) near event (or object) is the cause of the anxiety. Future anxiety also differs from those fears in which the physiological component of anxiety is in the focus. According to Zaleski [14], the objects of future anxiety are all events that can lead to a threat to the personal future. These threats can be socially relevant events, such as wars or natural disasters, as well as personally relevant events, such as health problems of one’s own or illness and loss of loved ones.

Expecting negative changes in the distant future may influence children’s and adolescents’ attitudes, decisions, and behaviors in the sense that they are likely to lower their expectations of positive outcomes of their own actions or that they avoid thinking about the future altogether [14, 15]. Under these circumstances they are less inclined to recognize opportunities because they are preoccupied by the risks which, in their view, strongly predominate. In a large longitudinal study, adolescents’ pessimistic perceptions of the future predicted depressive symptoms 20 to 30 years later in middle adulthood [16]. Although research on future related anxiety related to epidemics and other disasters is limited, theoretical and empirical reasons concur that anxious perceptions of the future may contribute to a restriction of educational, career, and life choices in youth [16, 17].

To our knowledge, future anxiety related to the ongoing COVID-19 pandemic has not been studied in primary school children, even though the pandemic has brought many changes to children’s families that could have a long-term impact on children’s well-being, such as parents losing their jobs, relatives dying or suffering from (Long-)COVID, or children being unable to make up for missed school lessons. In addition, primary school children oftentimes understand less about the COVID-19 virus than older age-groups and may therefore be particularly inclined to excessive or persistent anxiety during the pandemic [8, 18]. Therefore, this study aims to examine potential risk and resilience factors in children’s recovery from COVID-related future anxiety (CRFA) during the second year of the abating pandemic.

### Individual risk and resilience factors against CRFA

In a multisystem perspective of risk and resilience [19], resources against (future) anxiety can be found at the individual level because secure attachments, executive functions, optimism, and more active coping styles have been shown to increase resilience against stress [19] (Masten et al., 2021). In the longitudinal study by Hussong et al. [7] of initially 100 US children, youths with greater self-efficacy showed smaller increases in mental health symptoms during the first lockdown when compared to before. Problem-focused engaged coping also seemed to serve as a buffer against the rise in symptomatology during the first lockdown, whereas optimism, emotional coping and problem-focused disengaged coping did not serve as buffers [7]. In more general terms, emotion dysregulation seems to maintain (social) anxiety among children and adolescents. A review over 55 studies suggests that socially anxious children showed more avoidance, more safety behaviors, a bias in their attention and interpretation of social information, as well as repetitive negative thinking and reduced emotional expressions when compared to their agemates. Some of these emotion regulation strategies are likely to increase anxiety and none of them helps in reducing anxiety [20]. Therefore, not only adaptive emotion regulation strategies, such as problem-focused engaged coping, but also maladaptive strategies need to be considered when predicting children’s CRFA.

Another individual resilience factor [19] against CRFA may lie in children’s executive functions, i.e., “a family of top-down mental processes needed when you have to concentrate and pay attention, when going on automatic or relying on instinct or intuition would be ill-advised, insufficient, or impossible” [21]. Executive functions among children include (but are not limited to) the three components inhibitory control (or interference control), attentional flexibility, and updating (working memory updating; [21]). Working memory updating seems to be of foremost importance because it constitutes the basis for the other executive functions [21, 22]. Meta-analytic evidence among adults suggests that anxiety is likely to restrict the capacity of working memory by interfering with task-relevant processes [23]. Studies show that working memory updating tends to be impaired in anxious children [24]. However, the effect can be bidirectional. Because information processing in working memory is less effective in individuals with high levels of anxiety (due to interference), an efficient working memory may be an indicator for children’s ability to resist interferences resulting from anxiety or other forms of stress [25]. Having a well-developed working memory may thus help children “to shield themselves against” or “to fend off” the repetitive negative thoughts which are typical of (future) anxiety [17] and CRFA [26]. Thus, working memory updating should be included as a potential resilience factor in the longitudinal explanation of children’s CRFA.

### Background factors

*Gender* effects need to be considered because previous studies have shown that female youths have noted more future anxiety than their male agemates in general [17] and in relation to CRFA [18]. Female participants also reported more often that they suffered from symptoms of generalized anxiety during the COVID-19-pandemic [6], possibly because of loneliness [13] or lack of access to social support [27].

In order to account for systemic effects on risk and resilience [19], children’s families’ *socio-economic status* (SES) needs to be taken into account. Studies from various countries agree that children from families of a lower SES experienced more anxiety than children from more affluent families during the first lockdown [3, 8, 9]. Children from *immigrant families* often find themselves at the low end of the SES distribution [28]. These children were rated by their parents to have more total mental health problems (especially peer problems, but also more emotional problems) during the first lockdown than their agemates in Germany [9].

Having suffered from an *infection with the COVID-19 virus* themselves or having observed this illness in a family member, is also likely to increase children’s CRFA (in the months before the advent of vaccinations), because of the oftentimes severe symptoms, the concomitant quarantine, and the unknown long-term effects (Long COVID;[29, 30]). Thus, it is another background factor that needs to be controlled when predicting children’s level of CRFA.

### The current study

In this study 3^rd^- and 4^th^-graders were asked to self-report about their CRFA during regular classroom hours within a longitudinal intervention study. The present analyses focus on two measurement points in December 2020 and May 2021. They have two aims: 1) to explain children’s CRFA level in May 2021, and 2) to provide information about the development of children’s CRFA between December 2020 and May 2021. In so doing we aim to examine children’s psychological resilience to CRFA, with the expectation that children’s emotion regulation in December 2020 will help to explain their CRFA in May 2021. We also expect that children’s working memory will be a resilience factor which explains a low or decreasing level of their later CRFA. Both expectations are examined while controlling for background factors.

Hypotheses are:

1. Children’s CRFA in May 2021 is higher for children reporting maladaptive emotion regulation strategies and lower for children reporting adaptive emotion regulation strategies and performing better in a working memory updating task in December 2020 above and beyond social and personal predictors.
2. Children’s maladaptive emotion regulation contributes to an increase whereas their adaptive emotion regulation and their working memory updating performance contribute to a decrease of their CRFA from December 2020 to May 2021, above and beyond social and personal predictors.

## Methods

### Procedure

After obtaining approval for study from the Ethics Review Board of the authors’ university on May 6, 2020 that all research was performed in accordance with the relevant guidelines and regulations, the initial sample of the study was compiled in September 2020. The first data collection for the present analyses took place in December 2020. This was just before the second COVID-19-related lockdown in Germany. In May 2021 schools had just reopened, but children were in school only two or three days a week, because their classrooms had been split up into two groups to reduce infections. Children entered their data in tablets while sitting in class. Items and instructions were read aloud by a test administrator in the front of the classroom. The children could read along silently for themselves. Three to four additional trained undergraduate research assistants supported children with technical difficulties or insufficient computer skills in answering on the tablets. Our survey lasted three to four teaching hours at each measurement point and was interrupted by the usual breaks between the lessons. Children took part in the study only after they had brought the written consent of a parent or guardian. Children’s participation was voluntary. Children who were not allowed or did not want to participate were either supervised in another room or quietly occupied themselves with materials provided by the teacher. At the end of each data collection, all children received a small gift. Together with their consent form, parents completed a short questionnaire about their family situation, the language(s) spoken at home, and their educational and professional background.

Of the initial sample, *n* = 81 children from five classrooms were randomized into a breath-based mindfulness program which aimed at improving children’s attention, improving classroom climate, and increasing children’s performance in mathematics. It consisted of a selection of 15 short mindfulness-based breathing exercises which were led by teachers and performed over nine weeks. Due to this embedded intervention, we controlled for treatment exposure (coded as 0 = control and 1 = treatment) in subsequent analyses although no effects on children’s CRFA were found to distinguish the participating students from the *n* = 59 who had not participated.

### Measures

*COVID-related future anxiety*. Based on his theoretical work, Zaleski [14] developed and validated the Future Anxiety Scale, which measures adults’ anxiety about the future with a total of 29 items (including four positive filler items). The Future Anxiety Scale was condensed by [15] into five items and renamed Dark Future Scale. In 2020, the Dark Future Scale was translated into German and used with university students [31]. Within the course of the intervention study in which the present data were collected, the German Dark Future Scale was adapted to primary school children’s understanding by taking out one item and by focusing the questions more concretely on epidemics [32]. Children’s CRFA was assessed at both measurement points with the resulting Epidemic-Related Dark Future Scale for Children (eDFS-C; see S1 Table). The items of this scale are designed in a way that the name of a particular epidemic or pandemic, such as the COVID-19 pandemic, can be included in the items. The eDFS-C consists of four items which were scored on a four-point scale (0 = “never”, 1 = “seldom”, 2 = “sometimes”, 3 = “often”). No item is reverse scored. Raw scores can range from 0 to 12 with higher scores indicating more CRFA. An example item is “Are you afraid that your life may get worse because of the COVID-19 virus?” In the validation study with the present data, internal consistency was acceptable with α = .76 [32]. Comparable values were obtained in the study by Kästner et al. [18]. The eDFS-C overlaps partially but not completely with self-report measures of anxiety (*r* = .46 and *r* = .55) and teacher reports of emotional problems on the SDQ (*r* = .19) [32]). That children who had experienced the effects of the COVID-19 virus in their families (see below) tended to report higher levels of CRFA indicates criterion validity of the eDFS-C [32].

*Emotion regulation*. The short form of the German “Questionnaire for the Assessment of Emotion Regulation in Children and Adolescents” (FEEL K-J) [33, 34]was used to measure children’s self-reports of their emotion regulation in December 2020 and in May 2021. The original FEEL-KJ measures 15 emotion regulation strategies for the emotions fear, sadness, and anger, with 30 items for each emotion, thus 90 items for the total questionnaire. They are divided into the secondary scales *Adaptive strategies* (which are tallied over the strategy scales Problem oriented action, Distraction, Humor enhancement, Accepting, Forgetting, Cognitive problem solving, and Reappraisal), *Maladaptive strategies* (which are summed over the strategy scales Giving up, Aggressive behavior, Withdrawal, Self-deprecation, and Rumination), and *Other strategies* (Expression, Social Support, and Emotion Control*)*. Initially, the short form of the questionnaire was developed for parents’ report of emotion regulation [35]. The three emotions of the original form were combined into "unhappy", resulting in 30 items. The Adaptive strategies secondary scale of the short form included 14 items and the Maladaptive strategies secondary scale contained 10 items. The Other strategies secondary scale consisted of six items. Each strategy scale was represented by two items. The answers were Likert-type scaled (1 = “almost never”, 2 = “seldom”, 3 = “once in a while”, 4 = “oftentimes”, and 5 = “almost always”). Raw scores for the Adaptive strategies secondary scale could range from 14 to 70 and for the Maladaptive strategies secondary scale from 10 to 50 with higher scores indicating a more adaptive or maladaptive emotion regulation. For this self-report questionnaire, the wording of the item stems was: "When I am unhappy (sad, angry, anxious),…”. A sample item for a Maladaptive strategies secondary scale was “…, I keep thinking about why I am unhappy.” (Rumination). Internal consistency for the Maladaptive strategies secondary scale was acceptable with α = .70 in the present study and α = .82 in the norming sample of *N* = 780 children and adolescents. A sample item for the Adaptive strategies secondary scale was “…, I do something I enjoy.” Internal consistency for the Adaptive strategies secondary scale was good with α = .88 in the present sample. In a student’s thesis high correlations between the Adaptive strategies of the short version of the self-report FEEL-KJ and the *Target Congruent Scale* of a prefinal version of the Process-Oriented Emotion Regulation Measure for Children and Adolescents (POEM-CA) [36], *r* = .69) and between the Maladaptive Scale of the FEEL-KJ and the *Target Incongruent Scale* of the POEM-CA (*r* = .63)were found [37].

*Working memory updating*. Working memory updating was measured with an objective test in December 2020 and in May 2021. Children’s backward digit span was recorded on the tablet version of the EI-MAG [38]. A span of the digits 1 to 9 was audibly presented on the tablet via headphones, with a line interval of 1.5 seconds between each digit. Afterwards, a number block appeared on the screen with the digits 1 to 9. The children were instructed to tap the digits of the previously heard sequence in reverse order on the screen. Children’s correct answers and reaction times were automatically recorded. The EI-MAG is adaptive, i.e., the blocks of digit spans increase in difficulty. It starts with a block in which two digits need to be remembered. In the most difficult block, a span of eight digits must be reproduced. The test ends when children make three mistakes in a block.

*COVID in family*. Children’s experience of the effects of the COVID-19 virus in their family was examined in May 2021 with two self-constructed items which asked whether 1) the child himself or herself had been quarantined because of the COVID-19 virus and 2) at least one member of the child’s family had been quarantined because of the COVID-19 virus. If one or both questions were answered with "yes", it was counted as an experience of the COVID-19 virus within the family.

*Parent education*. The parents’ level of education was used as an indicator of the children’s socioeconomic status. The parents’ questionnaire was used to determine whether at least one parent in the child’s family had obtained a vocational qualification (= 1) or not (= 0). That is, whether they have completed a school-based or practical training that qualifies them to work in a profession.

*Migration background*. Children were considered to have a migration background if at least one of the following criteria applied to them: 1) both parents were born abroad, or 2) one parent and the child him- or herself were born abroad, or 3) the child grew up in a bilingual household. These data were obtained from the parents’ questionnaires.

### Statistical analyses

All statistical analyses were conducted with the software R [39]. For the main analyses the package *mice* [40] was used to perform multiple imputation by chained equations in order to deal with the proportion of about 5% missing data in the dataset so that the data of all *N* = 140 children of the initial sample could be included. Data were missing completely at random which was tested with Little’s Missing Completely at Random test [41]; *Χ^2^*(469) = 490.09, *p* = .242. Because [40] recommended using the amount of missing data as an indicator for the number of imputations, 5 imputations were used. All variables that were included in the analyses were used as predictors for the imputation process if the correlation with the other variables was at least *r* = .10. Missing values of all variables that were included in the analyses were imputed, with the exception for gender and age because there were no missing values for these variables. The first multiple linear regression analysis (Table 3) aimed at predicting children’s state of CRFA in May 2021. Children’s adaptive and maladaptive emotion regulation strategies as well as their working memory updating skills, all measured in December 2020, were included as main predictor variables, whereas gender, family migration background, group, and prior experience with COVID-19 quarantine in the family (in May 2021) were control variables. For the second multiple linear regression analysis (Table 4), CRFA in December 2020 was added as a predictor of CRFA in May 2021. When this score was controlled, the focus was on the development of CRFA over time and the role of emotion regulation and working memory updating as predictors of this development over time. Sensitivity analyses confirmed that there were no differences in the results when complete case analyses were performed.

## Results

### Sample

The initial sample of the study was recruited in September 2020 and included *N* = 140 children from *N* = 9 3^rd^ and 4^th^ grade classrooms in Northern Germany with *n* = 68 (49%) female participants. In December 2020, children’s age ranged between 8.16 and 11.41 with a mean of 9.16 years (*SD* = 8.12 months). In December 2020 eight children dropped out of the initial sample, but five children joined the study. In May 2021 seven additional children dropped out whereas six children who had not participated before joined the sample. Thus, sample sizes for December 2020 and May 2021 were *N* = 137 and *N* = 136, respectively. The reasons for missing measurements and drop-out were illness, moving to another city, and refusal to participate. Of the initial sample, 94% participated in the December 2020 and 93% in the May 2021 data collection.

Parents’ questionnaires were used to record the families’ migration history and their socioeconomic background in terms of the highest educational attainment within the family. The distributions of family characteristics are displayed in Table 1. Parents’ highest educational attainment could be calculated for *n* = 134 children. For *n* = 30 families (21%) no parent achieved a vocational qualification. In the initial sample, *n* = 31 children (25%) had one or two parents who were born abroad. Other indices of migration history were the country of birth of the children and multilingualism. The majority of children lived with both their parents and had already experienced COVID-19 in their families until May 2021.

**Table 1:** Distributions of characteristics of the children’s families.

	*n*	%
Parent’s educational attainment^a^		
No graduation	5	4
Secondary School Diploma^1^	18	13
A-Levels	7	5
Vocational Training	24	18
Technical College Degree	12	9
University Degree	68	51
Parents born in Germany^a^		
Both	100	75
One	16	13
None	15	12
Multilingualism^a^		
Yes	26	19
No	113	81
Child born in Germany^a^		
yes	126	91
no	12	9
Family situation^a^		
Both parents at home	117	84
Single mother	6	4
Single father	3	2
Single mother with new partner	10	7
Single father with new partner	0	0
Other situation	4	3
COVID-19 in family in May 2021^a^		
Yes	93	68
No	43	32

*Note*. ^1^and comparable degrees; ^a^ sums vary because of missing values

### Preliminary analyses

Before the main analyses data were screened for outliers and multicollinearity. Pearson correlations between variables and descriptive statistics are displayed in Table 2. Mean scores for CRFA showed that children scored about 4.5 points in December 2020 and 4.3 points in May 2021 with a range between 0 and 12 points. Changes in CRFA varied greatly between individuals. Between December 2020 and May 2021, CRFA decreased in 50% of children and increased in 35% of children. For around 10% of all children, there was an increase of 4 or more points between the two measurement points. In December 2020, 4% of the children reported not to have any CRFA, at all (0 points). This value increased to 12% in May 2021. However, the proportion of children who reported the highest score of 12 points remained relatively stable over the measurement points with 2% in December 2020 and 1.5% in May 2021. In December 2020, 14% of children stated "oftentimes" for at least one of the four items, in May 2021 it was 13% of the sample. Thus, overall CRFA was low in the sample, but some children experienced high CRFA. The slight decrease of children’s CRFA from December 2020 to May 2021 was not significant (*t*(254) = 0.989, *p* = .324), whereas the correlation between these two scores was (*r* = .60, *p* < .001).

**Table 2:** Pearson intercorrelations between variables and descriptive statistics.

	Dec20 Age in Months	Dec20 CRFA	May21 CRFA	Dec20 Adaptive ER	Dec20 Mal-adaptive ER	Dec20Work. Mem.
Dec20 CRFA	.16					
May21 CRFA	.15	.60***				
Dec20 Adaptive ER	.10	.10	.08			
Dec20 Maladaptive ER	.19*	.19*	.16	.21*		
Dec20 Working Memory	-.12	-.16	-.27**	.01	-.04	
Mean	109.96	4.53	4.22	37.62	24.97	3.64
SD	8.12	3.15	3.22	12.29	7.53	1.98
Range	98–137	0–12	0–12	14–66	10–45	0–8
Skew	0.96	0.55	0.64	0.08	0.27	-0.58
Kurtosis	0.77	-0.65	-0.43	-0.79	-0.37	-0.49

*Note*. CRFA = COVID-related Future Anxiety; Ad. = Adaptive; Malad. = maladaptive; ER = Emotion regulation; Work. Mem. = Working memory; *N* = 111–147

*** *p* < .001

Parent’s country of birth and children’s multilingualism were strongly associated (Mantel–Haenszel *Χ*^2^(1) = 57.424, *p <* .001, Cramer’s V = 0.84). Because migration history (as defined in the instruments section) and educational attainment were strongly associated (*Χ^2^*(1) = 31.244, *p* <. 001, Cramer’s V = 0.51), we chose to include only family migration in the following analyses. Group comparisons showed that CRFA in December 2020 (*t*(130) = -2.917, *p* = .004, *d* = 0.51) and in May 2021 (*t*(122) = -2.478, *p* = .015, *d* = 0.45) differed between boys and girls with girls reporting stronger CRFA. In addition, children’s CRFA was also stronger when parents reported no vocational attainment than when they had vocational attainment at both time points (December 2020: *t*(124) = -2.114, *p* = .036, *d* = 0.46; May 2021: *t*(116) = -3.662, *p* < .001, *d* = 0.81). Children who had experienced COVID-19 in their family until May 2021, reported stronger CRFA than children who had not experienced COVID-19 in their families (*t*(94) = -2.606, *p* = .011). Working memory updating skills in December 2020 did not correlate with adaptive or maladaptive emotion regulation but with CRFA in May 2021. Maladaptive regulation strategies significantly correlated with age, with CRFA in December 2020, and with adaptive regulation strategies.

### Regression analyses

The results of the first multiple regression analysis showed that girls and children with migration background reported to have more CRFA than boys and children without migration background (see Table 3). Above and beyond these control variables, children’s use of maladaptive emotion regulation strategies in December 2020 predicted higher CRFA scores in May 2021, whereas the use of adaptive strategies in December 2020 did not significantly affect CRFA in May 2021. Better working memory updating performance in December 2020 predicted lower CRFA scores in May 2021. This first model’s adjusted *R*^2^ was .27. Because adaptive strategies did not reach significance as a predictor in this model, the results were only partly in line with hypothesis 1.

**Table 3:** Prediction of children’s CRFA in May 2021 with linear regression analysis after multiple imputation.

	b	SE	t	*p*	95% CI
Intercept	0.345	1.345	0.256	.798	[-2.324, 3.013]
Gender (0 = male)	1.492	0.481	3.104	.023	[0.541, 2.444]
Family Migration (0 = no migration)	2.234	0.682	3.277	.018	[0.868, 3.599]
Treatment (0 = control group)	0.103	0.564	0.183	.856	[-1.023, 1.230]
May21 COVID in Family	0.997	0.601	1.659	.102	[-0.206, 2.200]
Dec20 Adaptive Emotion Regulation	-0.004	0.022	-0.204	.839	[-0.048, 0.030]
Dec20 Maladaptive Emotion Regulation	0.140	0.034	4.067	< .001	[0.072, 2.208]
Dec20 Working Memory	-0.388	0.149	-2.599	.016	[-0.697, -0.080]

*Note*. *N* = 140; CRFA = COVID-related future anxiety

When including children’s CRFA in December 2020 as a predictor, the second multiple linear regression analysis (in Table 4) showed that gender barely missed significance as a predictor of children’s CRFA, but migration status remained a predictor. CRFA in December 2020 was the strongest predictor of CRFA in May 2021. The positive effect of maladaptive emotion regulation strategies and the negative effect of working memory updating of the first model remained significant, whereas the effect of adaptive emotion regulation strategies was again not significant. The adjusted *R*^2^ was .43. Because the use adaptive strategies was not a significant predictor of CRFA in this model, the results of the regression analysis again only partially supported hypothesis 2.

**Table 4:** Prediction of children’s CRFA in May 2021 while controlling for CRFA in December 2020 with linear regression analysis after multiple imputation.

	b	SE	t	*p*	95% CI
Intercept	-0.209	1.234	-0.170	.866	[-2.669, 2.251]
Gender (0 = male)	0.846	0.438	1.931	.056	[-0.021, 1.713]
Family Migration (0 = no migration)	1.308	0.608	2.152	.035	[0.962, 2.519]
Treatment (0 = control group)	0.546	0.534	1.024	.313	[-0.534, 1.627]
May21 COVID in Family	0.538	0.528	1.019	.312	[-0.515, 1.591]
Dec20 CRFA	0.476	0.081	5.845	< .001	[0.314, 0.638]
Dec20 Adaptive Emotion Regulation	-0.004	0.019	-0.225	.822	[-0.041, 0.033]
Dec20 Maladaptive Emotion Regulation	0.084	0.031	2.696	.008	[0.022, 0.146]
Dec20 Working Memory	-0.299	0.140	-2.131	.047	[-0.594, -0.004]

*Note*. *N* = 140; CRFA = COVID-related future anxiety

## Discussion

Feeling anxious about a personal future which is rather uncertain in light of the largely uncontrollable COVID-19 pandemic in December 2020, is a rational response. It demonstrates primary school children’s increasing awareness of the perils that may restrict their chances in life. Mean values indicated a slight decrease of their CRFA during the second year of the pandemic, but with large interindividual differences. According to the ideas of Zaleski [14], levels of future anxiety can be explained by three factors: (1) subjective importance of the endangered value, (2) subjective probability of occurrence, and (3) controllability of the event. Health, family relationships, and friendships which were often strained by the COVID-19 pandemic were for children of paramount importance [3]. Because the mean incidence of COVID-19 per 100 000 inhabitants of the area of residence first rose and then declined, infections became less likely in May 2021. Controllability of COVID-19 infections grew because vaccinations had become available in the meantime. Mean levels of children’s CRFA may have decreased, according to Zaleski’s [14] analysis, because controllability increased, and infection rates abated towards May 2021.

### Predictors of CRFA in May 2021

Girls reported higher levels of CRFA than boys, both in December 2020 and in May 2021. This is in line with the study by Kästner et al. [18] which confirmed that females reported higher levels of CRFA. Additionally, a meta-analysis established that the prevalence rate of anxiety symptoms during the COVID-19 pandemic was significantly higher in samples with a higher proportion of female children and adolescents samples than in samples that included more boys [1]. Girls tend to feel more comfortable expressing anxiety than boys because it conforms more with a feminine gender role [42]. Other reasons may have to do with girls’ suffering more acutely from loneliness during periods of social isolation [13] and from higher exposure to violence and other adversity [6]. Therefore, the present results align with existing research on gender differences in anxiety symptoms and extend it to CRFA.

The effect of family migration on CRFA in May 2021 was significant as well. This may result from the fact that growing up in an immigrant family was associated with higher risks of infection over the whole period of the pandemic, because many immigrant families live in close quarters, which promotes infections. Immigrant parents also worked more often in jobs with much exposure to the public, such as nursing or sales [28]. Additional reasons for immigrant children’s elevated levels of anxiety may have to do with their parents’ lack of resources and a higher level of stress, e.g., because they had to work short hours, lost their jobs altogether, or because they had to educate their children in distance learning (in addition to their own job) although they themselves may not have had sufficient education for this task [28]. Experiencing COVID-19 within the family, which is likely to be associated with more intensive anxiety [19], seemed not to influence children’s CRFA in the present study. It is possible that the constant presence of the virus in the media and especially in children’s everyday lives was already enough to generate the children’s level of CRFA, so that a direct confrontation with the virus in one’s own family did not add to children’s CRFA.

When these family effects were controlled, children’s maladaptive emotion regulation in December 2020 explained their CRFA in May 2021 in a clear dose-response relation. The more children reported using maladaptive strategies such as Rumination (Perseveration), Withdrawal, Self-deprecation, and Giving up, the higher their levels of CRFA remained, despite the abating pandemic. Children who more often used maladaptive strategies of emotion regulation may have been like other anxious children in biasing their attention and interpretation of the available information on the pandemic to negative reports, and in repeatedly thinking about the pandemic [20]. Whether these well-known emotion regulation strategies, which tend to accompany depression and anxiety [43], have fueled children’s CRFA needs to be confirmed in more detailed studies in the future.

Children’s use of adaptive emotion regulation strategies seemed to have no effect on their CRFA in May 2021, neither on the level nor on the change over time. Given the actual (rather than the perceived) threat of COVID-19, using adaptive emotion regulation strategies might not have led to a decrease in anxiety, but merely to an appropriate management of the anxiety. For example, reappraising the pandemic would not have led children to conclude that there was no real threat or that the threat was previously considered to be too high. The role of adaptive emotion regulation strategies in a situation of real threat should also be investigated in more detail in further studies. The earlier mindfulness intervention did not show any effect because the breathing exercises addressed the physiological component of anxiety, and not the ruminative thoughts about the future. Besides, many children did not go to school on a regular basis between December 2020 and May 2021 because of the second lockdown.

As predicted, children’s working memory updating performance contributed to the prediction of their CRFA in May 2021 in both regression models. The higher their working memory updating performance was in December 2020, the more their CRFA was reduced five months later in May 2021. Having a well-functioning working memory updating thus seems to be a promotive resilience effect because it predicts better adjustment regardless of the level of the danger [19]. More specifically, managing anxiety requires a certain cognitive capacity. If there is less cognitive capacity, i.e., working memory updating, less can be spent on the adaptive regulation of anxiety, and anxiety remains higher [23, 44]. However, contrary to previous studies [45], neither maladaptive, nor adaptive emotion regulation skills were correlated with working memory updating performance in the present study. It needs to be investigated whether this results from a methodological bias, because working memory updating skills were assessed with a behavioral task and children’s emotion regulation skills were assessed with a self-report questionnaire.

Unsurprisingly, when adding CRFA in December 2020 to the prediction of CRFA on May 2021 in the second linear regression analysis, it explained the largest “chunk” of the variance of CRFA. Because earlier CRFA was included in the regression, children’s level of CRFA at a time in December 2020 in which infection rates increased dramatically was controlled. The effect of gender was not significant anymore, indicating that gender does not seem to influence the magnitude of increases or decreases in CRFA over time (slope), but only the state. However, the effects of maladaptive emotion regulation strategies and working memory updating remained significant, which supports their crucial roles as, respectively, risk and resilience factors for CRFA as discussed above.

Primary school children constitute a vulnerable group because many of them watch macro-level changes such as wars or pandemics on the media and react with future anxiety. Suffering from excessive or chronic anxiety can have a lasting impact on their mental health [46]. CRFA could be an early warning sign for the development of anxiety and depression in the years ahead [16]. The present study suggests that boosting the resilience factor working memory updating (and other executive functions; [47]) and reducing the risk factor repetitive negative thoughts (and other maladaptive strategies of emotion regulation) should be active ingredients of programs designed to prevent or alleviate children’s anxious reactions to future epidemics and related macro-level events. The proportion of children with lingering future anxiety furthermore underlines that grade schoolers are not immune to macro-level changes, but must be included in such measures of prevention and intervention in schools, clinics, families, and communities [27]. The newly devised eDFS-C scale which was presented in this paper may be used to document the success of these efforts.

### Strengths and limitations

Strengths of the current study include the examination of children’s self-reports of their CRFA over two measurement points which may provide more reliable information about their CRFA than reports by parents (or other adults) [48]. Conducting the study in primary school classrooms reduced the effects of social isolation (during the lockdowns) which tend to increase anxiety [13]. It also counteracts selection effects which can be observed in the volunteer samples of online studies. Another strength lies in the use of an objective measure of working memory updating. Limitations include the lack of well-established measures and the moderately sized sample from a rural region of Northern Germany with low incidence rates of COVID-19 during the study. Surely, results need to be replicated. A replication should be conducted with a more representative sample, over longer periods of time, and at different intensities of a pandemic. Interindividual differences may better be captured by person-centered approaches which elucidate different trajectories of the recovery from CRFA during a receding pandemic. Self-reports of CRFA and emotion regulation may be influenced by gender stereotypes and other concerns of self-presentation. Future studies should investigate further moderators of level and slope of children’s CRFA, such as their agency [17], their self-efficacy [7], and their social support within the family [4], classroom, or community, because “individual” resilience is usually linked to the resilience of other systems [19].

## Conclusion

The present study adds to the literature by confirming that maladaptive emotion regulation contributed to the prediction of children’s CRFA, a hitherto unknown type of anxiety, and that rumination etc. were detrimental in face of the real threat posed by the COVID-19 pandemic before the advent of vaccinations. Results also suggest that working memory updating may be a resilience factor in the face of this threat. These results apply to primary school children who are rarely studied. Among other individual resilience factors, less maladaptive emotion regulation and increased cognitive abilities contributed to the explanation of the level and change in primary school children’s CRFA during the abating COVID-19 pandemic in May 2021.

## Supporting information

S1 Table Longitudinal prediction of primary school children’s COVID-related future anxiety in the second year of the pandemic in Germany. (PDF)
