# Supplementary material for: Longitudinal prediction of primary school children’s COVID-related future anxiety in the second year of the pandemic in Germany
Source: PLoS One. 2024 May 8;19(5):e0302065. doi: 10.1371/journal.pone.0302065 (PMC11078412; doi:10.1371/journal.pone.0302065)
Supplement: S1 Table — (PDF) [file pone.0302065.s001.pdf]

SI Table 1. Dark Future Scale Short form for adults (Zaleski et al., 2019) adapted to children and to the COVID-19 pandemic

	COVID-related future anxiety (Voltmer & von Salisch, 2021)	Dark Future Scale Short Form (Zaleski et al., 2019)
1.	*Are you afraid, that the COVID-19 virus may stay on for a long time?*	I am afraid that the problems which trouble me now will continue for a long time
2.	----	I am terrified by the thought that I might sometimes face life’s crises or difficulties
3.	*Are you afraid that your life may get worse due to the COVID-19 virus?*	I am afraid that in the future my life will change for the worse
4.	*Are you afraid, that your family will soon be able to afford less because of the COVID-19 virus?*	I am afraid that changes in the economic and political situation will threaten my future
5.	*Are you afraid that the COVID-19 virus will prevent you from doing your hobbies, graduating from school, or doing your dream job in the future?*	I am disturbed by the thought that in the future I won’t be able to realize my goals
